# Inhibition Effects and Mechanism Study of rAj-HRP30, a Recombinant Histidine-Rich Peptide from *Apostichopus japonicus*, on the Viability of Pancreatic Ductal Adenocarcinoma Cells Panc01 and Panc02

**DOI:** 10.3390/ijms26041485

**Published:** 2025-02-11

**Authors:** Yuyao Song, Shan Gao, Jingwei Jiang, Yuebin Zhang, Jingyu Zhang, Xiaona Wang, Li Lv, Zunchun Zhou, Jihong Wang

**Affiliations:** 1 Ministry of Agriculture and Rural Affairs Key Lab of Protection and Utilization of Aquatic Germplasm Resource, Liaoning Province Key Lab of Germplasm Improvement and Fine Seed Breeding of Marine Aquatic Animals, Liaoning Ocean and Fisheries Science Research Institute, Dalian 116023, China; yolanda_songyy@hotmail.com (Y.S.); gs_070920@hotmail.com (S.G.); weijingjiang@live.cn (J.J.); 2 School of Life Sciences, Liaoning Normal University, Dalian 116081, China; yuebinz@hotmail.com (Y.Z.); jingyuzhang2000@hotmail.com (J.Z.); wangxiaonalg@hotmail.com (X.W.); 3 Department of Pharmacology, College of Pharmacy, Dalian Medical University, Dalian 116044, China; lvli@dmu.edu.cn

**Keywords:** histidine-rich peptide, Panc01, Panc02, antitumor peptide, FGFR1

## Abstract

rAj-HRP30 is a recombinant peptide derived from the wild-type rAj-HRP of *Apostichopus japonicus* through a gene-shortening mutation. It has a high histidine content (53.3% in its primary structure) and a molecular weight of 3.919 kDa, classifying it as a histidine-rich peptide. The literature reports indicate that human histidine-rich peptides exhibit antitumor activity. Previous research by our group demonstrated similar properties in rAj-HRP, the precursor of rAj-HRP30. Therefore, this study used Panc01 (human) and Panc02 (mouse) cells—highly malignant models with limited targeted therapies—to investigate the antitumor activity and mechanisms of rAj-HRP30 and evaluate its potential for pancreatic cancer treatment. This study designed a gene-shortening strategy for rAj-HRP and artificially synthesized the gene sequence of rAj-HRP30. The cDNA sequence of rAj-HRP30 was cloned into the pET23b vector, and the recombinant plasmid pET23b-HRP30 was transformed into *E. coli* BL21 for expression. Following IPTG induction, the recombinant peptide was purified using nickel ion affinity chromatography, yielding rAj-HRP30 with a purity exceeding 95%. rAj-HRP30 markedly inhibited the adhesion, migration, and invasion of Panc01 and Panc02 cells. It also disrupted cellular morphology and cytoskeletal structure while inducing apoptosis. These effects were dose-dependent. After confirming the in vitro anticancer activity of rAj-HRP30, this study employed Panc02 cells as a model to investigate its inhibitory mechanisms using Western blot analysis. The results revealed that rAj-HRP30 reduced FGFR1 expression in Panc02 cells and inhibited the downstream FYN and FAK signaling pathways, subsequently blocking the PI3K/AKT signaling and apoptosis pathways. In the apoptotic pathway, rAj-HRP30 was able to downregulate the expression of Bcl-2, Caspase-9, Caspase-3, Caspase-7, and PARP1 and upregulate the expression of Bax, cleaved Caspase-9, cleaved Caspase-3, cleaved Caspase-7, and cleaved-PARP1 to induce apoptosis in Panc02 cells. Furthermore, rAj-HRP30 also downregulated the expression of MMP2 and MMP9, thereby inhibiting the migration and invasion of Panc02 cells. Conclusion: rAj-HRP30 exhibits significant inhibitory effects on pancreatic ductal adenocarcinoma Panc01 and Panc02 cells in vitro. Its mechanism involves FGFR1-related signaling and apoptosis pathways. rAj-HRP30 shows promise as a therapeutic agent targeting FGFR for pancreatic cancer.

## 1. Introduction

In 2020, the global incidence of cancer reached 19.29 million new cases. Pancreatic cancer is among the most lethal malignancies, primarily due to its exceptionally poor prognosis. The five-year survival rate for pancreatic cancer typically remains below 15%. Unlike most other malignancies, pancreatic cancer shows rising trends in both incidence and mortality. Key modifiable risk factors include smoking, obesity, diabetes, and alcohol intake, which contribute to the rising incidence rates, while inherited genetic factors, including mutations in cancer-related and pancreatitis genes, also affect the risk [1]. By 2030, pancreatic cancer is projected to become the second leading cause of cancer-related deaths in the United States, second only to lung cancer. The burden of pancreatic cancer is expected to rise further due to an aging population and increasing life expectancy [2]. Current treatment strategies for pancreatic cancer include surgical intervention, pharmacotherapy, radiotherapy, molecular targeted therapy, cancer stem cell therapy, and emerging biotechnologies. Early detection and surgical resection offer the best chance for curing this cancer; however, most patients present with an advanced unresectable disease. The identification of molecular therapeutic targets to increase the efficacy of treatment regimens offers the potential to improve survival [3].

Currently, the research on pancreatic cancer treatment is primarily focused on developing new molecular targeted therapies. The targeted therapies currently used for pancreatic cancer include erlotinib, trastuzumab, trametinib, and bevacizumab. These therapies act on specific molecular targets: erlotinib targets the epidermal growth factor receptor (EGFR) [4], trastuzumab targets HER2 [5], trametinib targets mitogen-activated protein kinase (MAPK) [6], and bevacizumab targets vascular endothelial growth factor (VEGF) [7]. Most of these drugs were originally developed for other cancers but have also been tested for pancreatic cancer treatment. It has been shown that the inhibition of FGF/FGFR signaling decreases SOX2 expression, which impairs the sphere-forming ability and tumorigenic potential of PDAC cells. The overexpression of SOX2 in SOX2-negative cells induces sphere formation, and the inhibition of AKT signaling further disrupts stemness by reducing SOX2 nuclear expression [8]. In addition to this, fibroblast growth factor receptor 1 (FGFR1) was found to be a key driver of gemcitabine resistance (GemR) and increased tumor aggressiveness. The knockdown of FGFR1 or combination therapy with a selective FGFR inhibitor (FGFRi) enhances the efficacy of gemcitabine and reduces the GemR markers, making FGFR1 a potential therapeutic target for overcoming chemotherapy resistance and improving PDAC outcomes [9].

Oceans, with their unique and complex ecosystems, have a rich biodiversity. As biotechnology advances, the amount of research on bioactive marine substances is steadily increasing. Among numerous other bioactive marine substances, the discovery of endogenous peptide activity has made peptides the primary potential resource for these substances [10]. Malignant tumors are a major global health threat, affecting both China and the world. The ocean is rich in a large number of antitumor agents and natural products, which may offer new targeted therapies for cancer treatment. Guzmán et al. isolated Theopederins K and Theopederins L from deep-water sponges (*Discodermia*) and Mycalamide A from *Mycala*. All three molecules inhibited interleukin-8 (IL-8) secretion in several pancreatic cancer cell lines [11]. Several compounds obtained from marine molluscs, such as alkaloids, carotenoids, and shell toxins, have been shown to have inhibitory effects in a variety of cancers over the years [12]. Bryophytes are organisms with unique biological structures and are thereby a source of a wide range of bioactive molecules such as alkaloids and polyketides [13]. Macroalgae, also known as seaweeds, are photosynthesis organisms that are classified as brown (Ochrophyta), green (Chlorophyta), or red algae (Rhodophyta) [14]. Fucoidan, a sulphated polysaccharide, has been extracted from brown algae, and many studies have shown that it possesses a variety of biological activities, such as antitumor, anti-inflammatory, anti-oxidant, and anti-angiogenic properties [15,16,17]. Delma et al. found that fucoidan extracted from *Turbinaria conoides* had an inhibitory effect on different pancreatic cancer cell lines, inhibiting cell proliferation and causing apoptosis of pancreatic cancer cells by inducing the activation of Caspase-3, -8, and -9 [18].

In recent years, with the continuous research and development of antitumor drugs, peptides have gradually come into the public’s view, bringing new opportunities and challenges for the treatment of pancreatic cancer. With their advantages of a small molecular weight, weak antigenicity, strong targeting, and simple and environmentally friendly synthesis, antitumor peptides have shown great potential in clinical applications for the treatment of many malignant tumors, including pancreatic tumors. In previous studies, we identified and cloned a histidine-rich peptide, rAj-HRP, from the cDNA library of *Apostichopus japonicus* and confirmed its significant antitumor activity in vitro and in vivo. Studies have shown that rAj-HRP inhibits the ex vivo activity of human colon cancer HCT116 cells by acting on both the EGFR signaling and apoptosis pathways, targeting EGFR [19]. To avoid potential immunogenicity risks, it is necessary to further shorten and modify the rAj-HRP sequence. After bioinformatics analysis, we designed a mutation scheme to shorten the rAj-HRP sequence, then synthesized, cloned, expressed, and purified the shortened rAj-HRP gene. Because the new mutation is composed of 30 amino acids rich in histidine, it is named histidine-rich peptide rAj-HRP30. Bioinformatics analysis showed that rAj-HRP30 shares over 60% similarity with histidine-rich glycoprotein-like (HRG-like) sequences of species such as *Spodoptera litura* and *Plasmodium lophurae*. Although the functions of HRG-like proteins in these species remain unclear, the biological functions of human histidine-rich glycoprotein (HRG) have been well studied. Human HRG is a multi-domain plasma glycoprotein that interacts with Zn^2+^, pro-myosin, heparin, heparan sulfate, fibrinogen, and other ligands to exert various biological functions [20], including neutralizing the anticoagulant effect of heparin, binding divalent cations and heparin, removing immune complexes, and regulating tumor angiogenesis and immune responses [21,22]. Among these functions, the most noteworthy is its role in inhibiting tumor growth and metastasis [23]. Since it has been confirmed that the precursor of rAj-HRP30, rAj-HRP, has an antitumor function, we need to identify whether rAj-HRP30 has the same ability [19]. As there is currently no effective treatment for human pancreatic cancer, we plan to study the anti-pancreatic cancer function and mechanism of rAj-HRP30 using pancreatic duct cancer Panc01 (human origin) and Panc02 (mouse origin) cells as models to evaluate whether it has the potential to become an anti-pancreatic cancer drug.

## 2. Results

### 2.1. Molecular Cloning and Bioinformatics Analysis of rAj-HRP30

#### 2.1.1. rAj-HRP30 Is a Genetically Modified Peptide Derived from rAj-HRP

**Figure 1:**
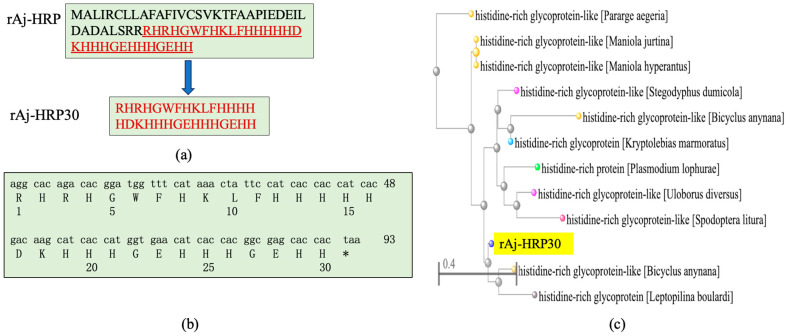
(**a**) rAj-HRP30 is a truncated peptide derived from the wild-type rAj-HRP, with the amino acid sequence of rAj-HRP30 highlighted in red underline. (**b**) The artificially synthesized cDNA sequence of rAj-HRP and its deduced amino acid sequence, * represents the termination codon. (**c**) Phylogenetic tree analysis of rAj-HRP30 (with rAj-HRP30 highlighted in yellow).

After analyzing the rAj-HRP sequence, we selected the amino acid sequence from positions 38 to 67 as the core sequence of rAj-HRP30 (Figure 1a). After the gene was optimized for better compatibility with the *E. coli* expression system, the cDNA sequence was finalized and artificially synthesized. Due to the fact that protein drugs are not allowed to carry purification tags, a stop codon was added to the 3′ end of the rAj-HRP30 cDNA. The synthetic cDNA sequence of rAj-HRP30 and its deduced amino acid sequence are shown in Figure 1b. Sequence alignment of rAj-HRP30 revealed that rAj-HRP30 shares over 68% sequence identity with histidine-rich glycoprotein-like (HRG-like) proteins from species such as *Spodoptera litura*, *Plasmodium lophurae*, *Bicyclus anynana*, and *Pararge aegeria* (Figure 1c).

#### 2.1.2. The Synthetic cDNA of rAj-HRP30 Is Inserted into the pET23b Vector

**Figure 2:**
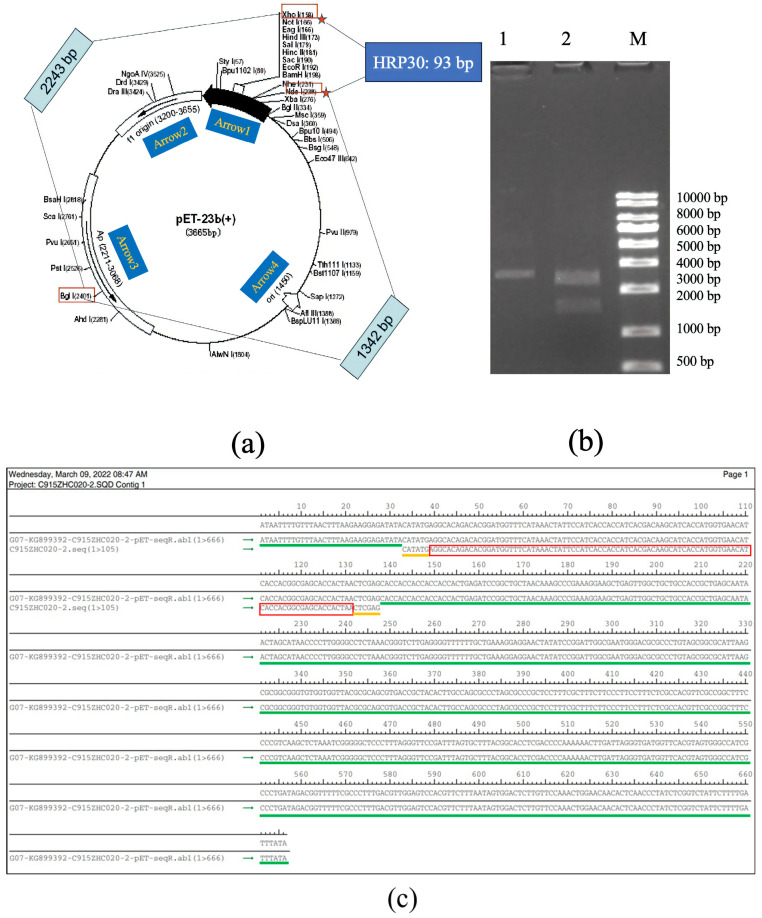
(**a**) Schematic representation of the pET23b vector containing the rAj-HRP30 gene and the restriction sites identified through double digestion. Arrow 1 represents the insertion direction of the multiple cloning sites (MCS); Arrow 2 represents the copy direction of f1 origin; Arrow 3 represents the transcription direction of the resistance gene Amp; Arrow 4 represents the copy direction of ori. (**b**) Restriction enzyme digestion analysis of the pET23b-HRP30 plasmid: approximately 200–1000 ng of plasmid DNA was digested at 37 °C for 30–60 min and analyzed on a 1% agarose gel. Lane M: DNA size marker (KB ladder); Lane 1: undigested pET23b-HRP30 recombinant plasmid; Lane 2: pET23b-HRP30 recombinant plasmid digested with *Bgl* I and *Xho* I (This data is from Kingsley Biotechnology (Nanjing, China)). (**c**) Sequencing results of the pET23b-HRP30 recombinant plasmid: The green marker represents the DNA sequence of pET23b plasmid. The yellow markers represent the *Nde* I and *Xho* I restriction enzyme sites. The red marker represents the cDNA sequence of HRP30.

The synthetic cDNA of rAj-HRP30 was inserted into the pET23b vector using the *Nde* I and *Xho* I restriction enzyme sites. The plasmid was extracted from positive bacterial colonies and double-digested with *Bgl* I and *Xho* I for confirmation. The double digestion analysis revealed that the recombinant plasmid products were 2243 and 1435 bp DNA fragments, confirming successful plasmid construction (Figure 2a,b). DNA sequencing of the recombinant plasmid confirmed that the results match the cDNA sequence of the rAj-HRP30 we designed (Figure 2c), verifying the correct construction of the pET23b-HRP30 recombinant plasmid.

### 2.2. Induction Expression and Purification Results of rAj-HRP30

**Figure 3:**
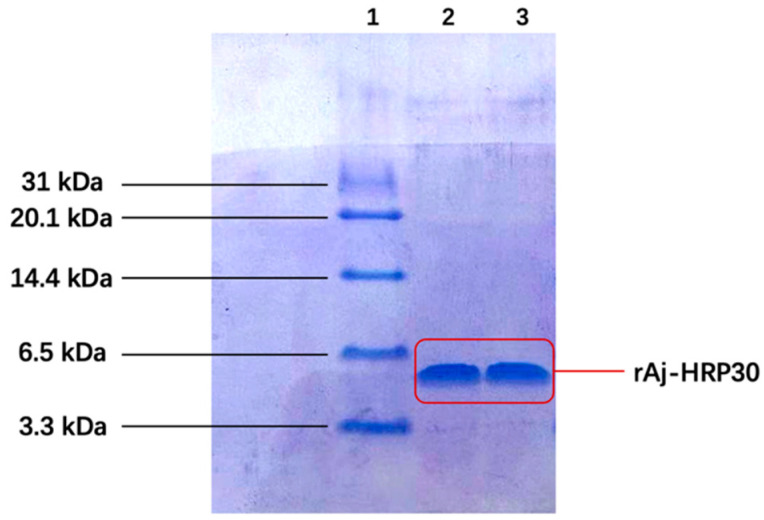
The tricine SDS-PAGE results of rAj-HRP30 purification. Lane 1: marker; Lane 2: purified rAj-HRP30; Lane 3: purified rAj-HRP30.

Following IPTG induction, the recombinant peptide was successfully purified. The primary sequence of rAj-HRP30 consists of 30 amino acids, 16 of which are histidine (Figure 1a). This histidine-rich sequence was used for purification via nickel ion affinity chromatography. Three-dimensional SDS-PAGE analysis confirmed that the purified recombinant peptide rAj-HRP30 was obtained with a purity exceeding 95% (Figure 3). Furthermore, the N-terminal sequencing results further validated the accuracy of the rAj-HRP30 expression sequence.

### 2.3. rAj-HRP30 Inhibits the Proliferation of Panc01 and Panc02 Cells

**Figure 4:**
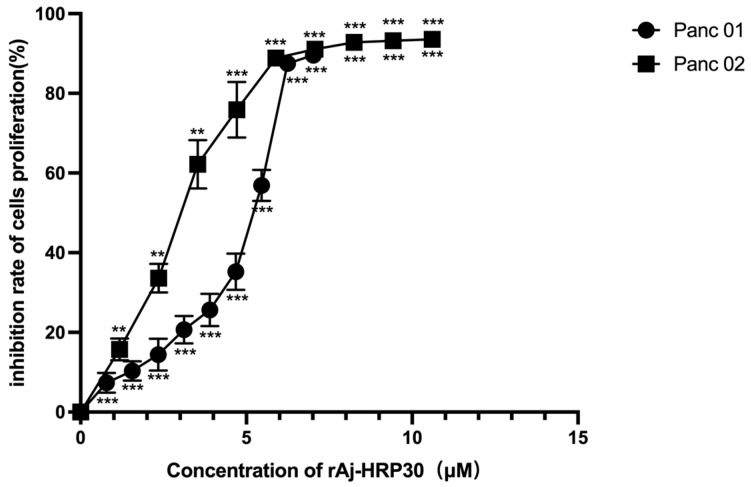
The effect of rAj-HRP30 on Panc01 and Panc02 cell proliferation was determined using the CCK-8 method (*n* = 3). The control group was treated with a blank medium, and the experimental group was treated with rAj-HRP30. Significant difference between the blank group and each experimental group (** *p* < 0.01, and *** *p* < 0.001).

We employed a CCK-8 assay to evaluate the effect of rAj-HRP30 on the proliferation of Panc01 and Panc02 cells. The results (Figure 4) show that rAj-HRP30 significantly inhibited the growth of Panc01 and Panc02 cells in a concentration-dependent manner. These findings confirm that rAj-HRP30 exerts a concentration-dependent inhibitory effect on the proliferation of Panc01 and Panc02 cells. A data analysis using GraphPad Prism 8.0 yielded IC_50_ values of 5.54 and 3.22 μM for rAj-HRP30 against Panc01 and Panc02 cells, respectively. These results provide a basis for determining the dosing regimen in subsequent experiments.

### 2.4. rAj-HRP30 Disrupts the Morphology of Panc01 and Panc02 Cells

**Figure 5:**
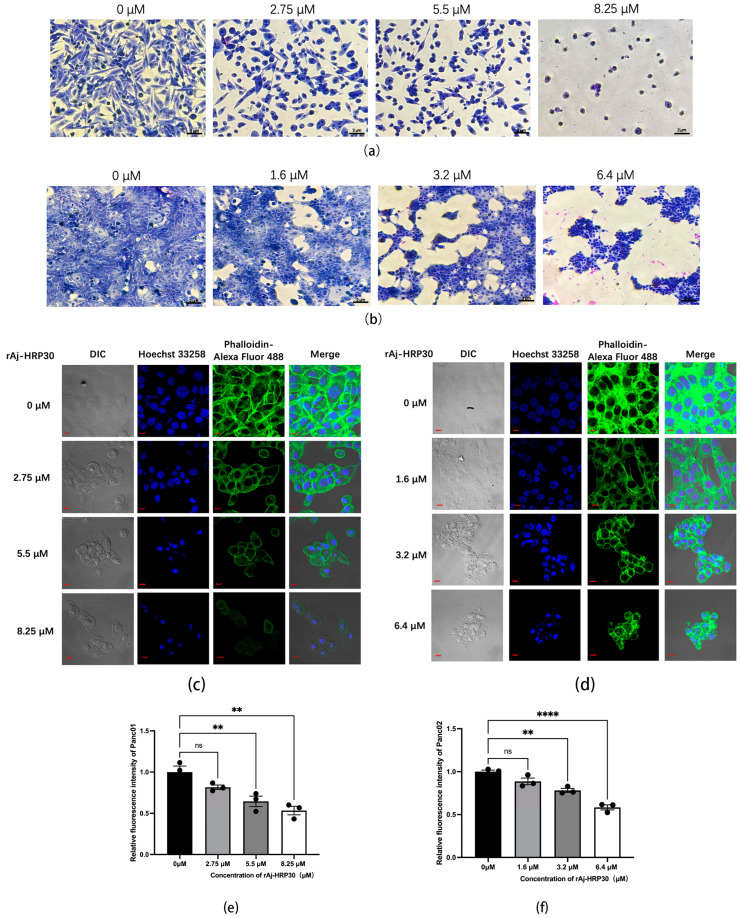
(**a**) Effect of rAj-HRP30 on the morphology of Panc01 cells (OLYMPUS digital imaging microscope, Tokyo, Japan, 200×; scale: 2 μm). (**b**) Effect of rAj-HRP30 on the morphology of Panc02 cells (OLYMPUS digital imaging microscope, Tokyo, Japan, 200×; scale: 2 μm). (**c**) The effects of rAj-HRP30 on the cytoskeleton and nucleus of Panc01 were determined using an FITC-ghost cyclic peptide assay (Zeiss laser scanning confocal microscope, Oberkochen, Baden-Wurttemberg, Germany, 200×; scale: 2 μm). (**d**) The effects of rAj-HRP30 on the cytoskeleton and nucleus of Panc02 were determined using an FITC-ghost cyclic peptide assay (Zeiss laser scanning confocal microscope, Oberkochen, Baden-Wurttemberg, Germany,200×; scale: 2 μm). (**e**) Effect of rAj-HRP30 on changes in relative fluorescence intensity of the Panc01 cytoskeleton (*n* = 3). The control group was treated with a blank medium, and the experimental group was treated with rAj-HRP30. ns indicates no statistical significance; ** indicates significant difference between the blank group and each experimental group (** *p* < 0.01). (**f**) Effect of rAj-HRP30 on changes in relative fluorescence intensity of the Panc02 cytoskeleton (*n* = 3). The control group was treated with a blank medium, and the experimental group was treated with rAj-HRP30. ns indicates no statistical significance; **, **** indicates significant difference between the blank group and each experimental group (** *p* < 0.01 and **** *p* < 0.0001).

Giemsa staining was employed to assess the morphological effects of rAj-HRP30 on Panc01 and Panc02 cells. The experimental results (Figure 5a,b) revealed that, with increasing concentrations of rAj-HRP30, there was a progressive reduction in both the cell density and intercellular connections. Concurrently, the cells exhibited signs of shrinkage, rounding, and detachment, while cytoplasmic condensation, membrane retraction, nuclear envelope rupture, and chromatin fragmentation into clumps were also observed. These morphological alterations indicate that rAj-HRP30 induces apoptosis in Panc01 and Panc02 cells, with its effects being concentration-dependent.

This study employed Alexa Fluor 488 Phalloidin staining to investigate the effect of rAj-HRP30 on the cytoskeletal architecture of Panc01 and Panc02 cells. The experimental results (Figure 5c,d) demonstrated that, in the control group, untreated cells displayed a well-defined fibrous cytoskeletal structure with clear contours and intact architecture. As the concentration of rAj-HRP30 gradually increased, the intercellular connections diminished progressively, and the microfilament networks within the cytoskeleton were disrupted, leading to a gradual rounding of the cells. In the high-dose group, the cytoskeletal structure was almost entirely compromised, with cells aggregating and cellular debris becoming conspicuous. These findings indicate that rAj-HRP30 significantly impairs the cytoskeletal integrity of both Panc01 and Panc02 cells in a concentration-dependent manner, thereby inducing apoptosis and further inhibiting critical cellular processes, including proliferation, adhesion, and migration.

### 2.5. rAj-HRP30 Inhibits the Migration and Invasion of Panc01 and Panc02 Cells

**Figure 6:**
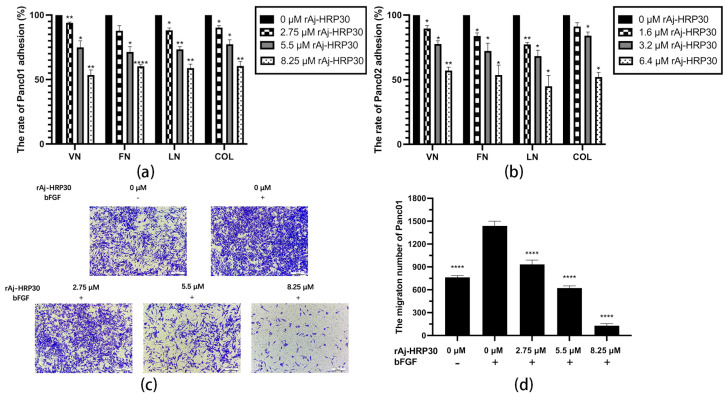

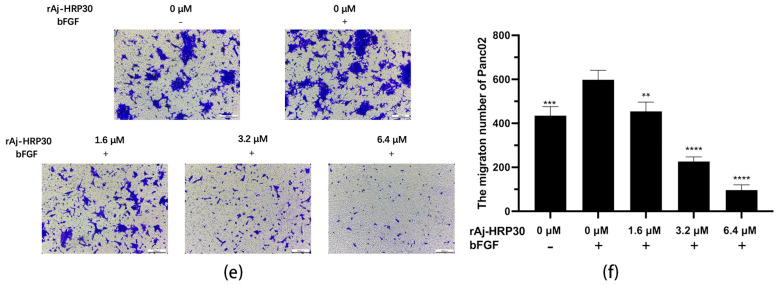
(**a**) The effect of rAj-HRP30 on the adhesion of Panc01 cells was determined (*n* = 3); significant differences between the experimental group treated with rAj-HRP30 and the control group treated with empty medium are indicated by * *p* < 0.05, ** *p* < 0.01, and **** *p* < 0.0001. (**b**) The effect of rAj-HRP30 on the adhesion of Panc02 cells was determined (*n* = 3); significant differences between the experimental group treated with rAj-HRP30 and the control group treated with empty medium are indicated by * *p* < 0.05, ** *p* < 0.01. (**c**) The degree of Panc01 cell migration under different administered concentrations (OLYMPUS digital imaging microscope, scale: 200 μm). (**d**) The amount of Panc01 cell migration under different administered concentrations (*n* = 3). The experimental group was treated with bFGF- or rAj-HRP30, and the control group was treated with bFGF+ without medication. Significant differences are expressed by **** *p* < 0.0001. (**e**) The degree of Panc02 cell migration under different administered concentrations (OLYMPUS digital imaging microscope, scale: 200 μm). (**f**) The amount of Panc02 cell migration under different administered concentrations (*n* = 3). The experimental group was treated with bFGF- or rAj-HRP30, and the control group was treated with bFGF+ without medication. Significant differences are expressed by ** *p* < 0.01, *** *p* < 0.001, and **** *p* < 0.0001.

In this study, collagen (COL), vitronectin (VN), laminin (LN), and fibronectin (FN) were selected as the primary ECM components to investigate the effects of varying concentrations of rAj-HRP30 on the adhesion of Panc01 and Panc02 cells. The experimental results (Figure 6a,b) demonstrate that as the concentration of rAj-HRP30 increased, the adhesion rate of cells to these four ECM proteins gradually decreased. These findings indicate that rAj-HRP30 significantly inhibits the adhesion of Panc01 and Panc02 cells to ECM proteins, with a concentration-dependent effect.

Previous experiments have demonstrated that rAj-HRP30 significantly inhibits the adhesion of pancreatic cancer cells to extracellular matrix components. In the present study, Transwell migration assay plates (Corning Incorporated, Corning, New York, NY, USA) were employed to replicate the three-dimensional microenvironment of tumors in vivo, enabling the evaluation of rAj-HRP30’s effects on the migration capacity of pancreatic cancer cells. The experimental results (Figure 6c–f) indicated that, in the control group with bFGF added to the lower chamber, the number of migrating cells was significantly greater than in the control group without bFGF. This suggests that the chemotactic factor bFGF effectively promotes cell migration. Compared with the bFGF-treated control group, a dose-dependent decrease in the migration of Panc01 and Panc02 cells was observed as the concentration of rAj-HRP30 increased, further confirming that rAj-HRP30 inhibits the migratory ability of pancreatic cancer cells in a concentration-dependent manner.

**Figure 7:**
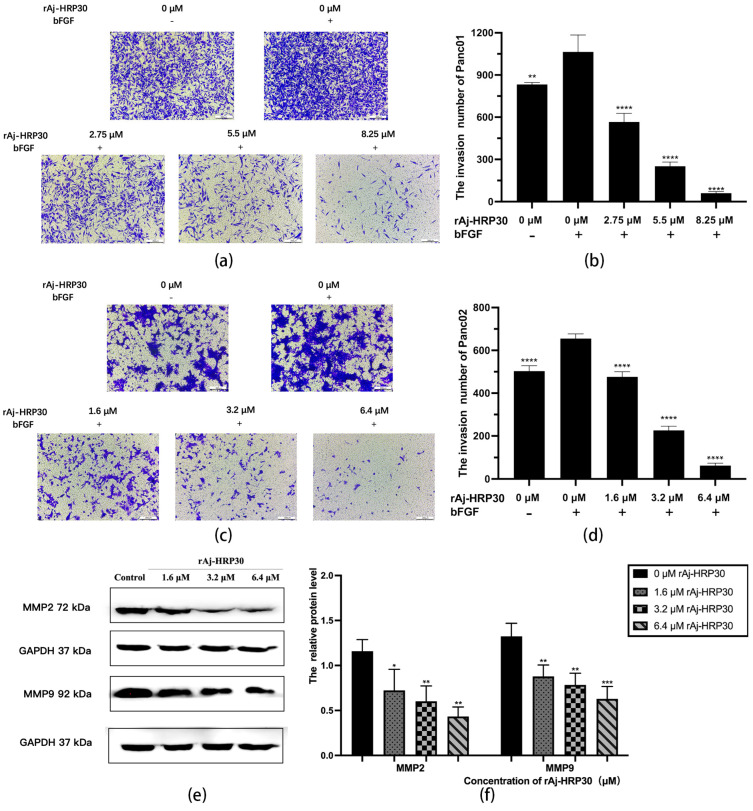
(**a**) Representative results of Panc01 cell invasion in each group (OLYMPUS digital imaging microscope, scale: 200 μm). (**b**) Statistics on the number of Panc01 cells invaded in each group (*n* = 3); significant differences between the experimental group treated with bFGF- or rAj-HRP30 and the control group treated with bFGF are indicated by ** *p* < 0.01 and **** *p* < 0.0001. (**c**) Representative results of Panc02 cell invasion in each group (OLYMPUS digital imaging microscope, scale: 200 μm). (**d**) Statistics on the number of Panc02 cells invaded in each group (*n* = 3); significant differences between the experimental group treated with bFGF- or rAj-HRP30 and the control group treated with bFGF are expressed by **** *p* < 0.0001. (**e**) Bar graph illustrating the Western blot results for MMP2 and MMP9 expression in Panc02 cells treated with rAj-HRP30. (**f**) Statistical analysis of the relative optical densities of MMP2 and MMP9 (target proteins) normalized to GAPDH levels (*n* = 3). The control group was treated with a blank medium, and the experimental groups were treated with rAj-HRP30. Significant differences between the blank group and the experimental groups are expressed by * *p* < 0.05, ** *p* < 0.01, *** *p* < 0.001.

To simulate the in vivo three-dimensional environment, this study utilized Transwell chambers with polycarbonate membranes coated with the artificial ECM matrix Matrigel. The results (Figure 7a–d) reveal that, compared with the control group without bFGF, the cell invasion rate was significantly higher in the group with bFGF in the lower chamber, indicating that the chemokine bFGF effectively promotes the invasion of Panc01 and Panc02 cells. Using the bFGF-treated non-drug group as a control, the results further demonstrate that, with increasing concentrations of rAj-HRP30, the number of invading cells gradually decreased, thereby providing compelling evidence that rAj-HRP30 effectively inhibits the bFGF-induced invasion of Panc01 and Panc02 cells, and this inhibitory effect is concentration-dependent.

Matrix metalloproteinases (MMPs) are aberrantly expressed in numerous cancer types and participate in critical pathological processes such as tumor initiation, progression, invasion, and metastasis by modulating signaling pathways and the tumor microenvironment [24]. In this experiment, Panc02 cells were treated with different concentrations of rAj-HRP30 to determine its effect on the expression of MMP2 and MMP9 in these cells. The experimental results (Figure 7e,f) demonstrate a dose-dependent decrease in the relative expression levels of MMP2 and MMP9 as the concentration of rAj-HRP30 increased, indicating that rAj-HRP30 downregulates the expression of MMP2 and MMP9. This suppression, in turn, inhibited the corresponding signaling pathways, ultimately impairing the migratory capacity of Panc02 cells.

### 2.6. rAj-HRP30 Induces Apoptosis in Panc01 and Panc02 Cells

**Figure 8:**
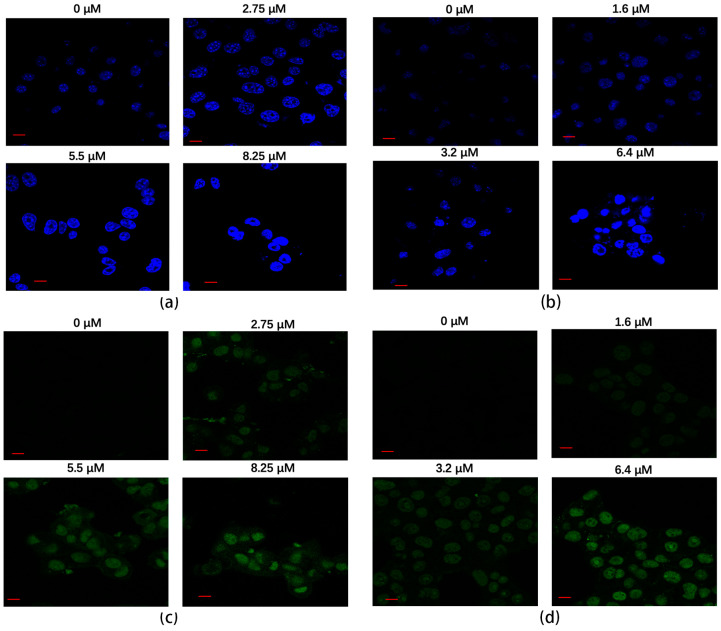
(**a**) The apoptotic effect of rAj-HRP30 on Panc01 cells was determined using the Hoechst method (Zeiss laser scanning confocal microscope, 630×; scale: 10 μm). (**b**) The apoptotic effect of rAj-HRP30 on Panc02 cells was determined using the Hoechst method (Zeiss laser scanning confocal microscope, 630×; scale: 10 μm). (**c**) The apoptotic effect of rAj-HRP30 on Panc01 cells was determined using the TUNEL method (Zeiss laser scanning confocal microscope, 630×; scale: 10 μm). (**d**) The apoptotic effect of rAj-HRP30 on Panc02 cells was determined using the TUNEL method (Zeiss laser scanning confocal microscope, 630×; scale: 10 μm).

The Hoechst staining method was employed to assess the apoptotic effects of rAj-HRP30 on Panc01 and Panc02 cells. The results (Figure 8a,b) reveal that, in the untreated control groups, the cell nuclei exhibited a pale color with deeper blue granules present within the nucleus, and the cells maintained a relatively regular near-spherical shape. With increasing concentrations of rAj-HRP30, the nuclear staining progressively lightened, accompanied by a noticeable aggregation of nuclear material. In the high-dose treatment group, fluorescent signals of DNA fragmentation were also observed. These findings collectively indicate that rAj-HRP30 induces apoptosis in both Panc01 and Panc02 cells, and the apoptotic effect is dose-dependent, intensifying with higher concentrations of the drug.

A TUNEL assay was employed to assess the effect of rAj-HRP30 on apoptosis in Panc01 and Panc02 cells. The results (Figure 8c,d) reveal that cells in the untreated control groups exhibited minimal green fluorescence, indicating a lack of significant DNA fragmentation. However, with increasing concentrations of rAj-HRP30, the green fluorescence signal progressively intensified, accompanied by a decrease in cell numbers. Collectively, these findings suggest that rAj-HRP30 effectively induces apoptosis in Panc01 and Panc02 cells, with the apoptotic effect becoming more pronounced in a dose-dependent manner.

**Figure 9:**
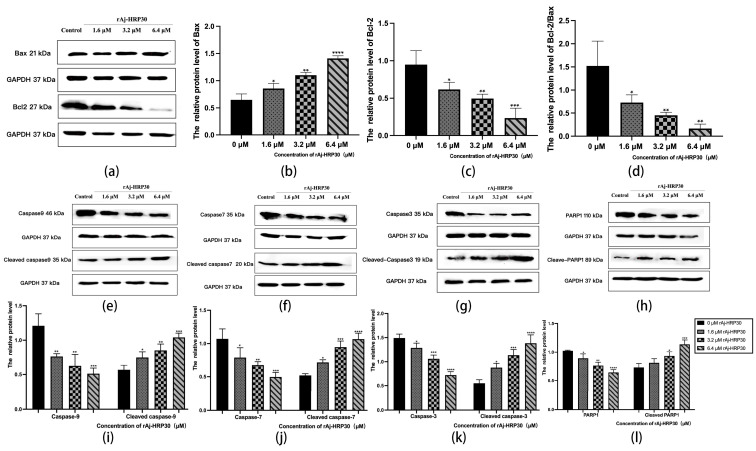
(**a**) Bar graph illustrating the Western blot results for Bax and Bcl-2 expression in Panc02 cells treated with rAj-HRP30. (**b**) Statistical analysis of the relative optical densities of Bax (target proteins) normalized to GAPDH levels. (**c**) Statistical analysis of the relative optical densities of Bcl-2 (target proteins) normalized to GAPDH levels. (**d**) Statistical analysis of the relative optical densities of Bcl-2/Bax (target proteins) normalized to GAPDH levels. (**e**) Bar graph illustrating the Western blot results for Caspase-9 and cleaved Caspase-9 expression in Panc02 cells treated with rAj-HRP30. (**f**) Bar graph illustrating the Western blot results for Caspase-7 and cleaved Caspase-7 expression in Panc02 cells treated with rAj-HRP30. (**g**) Bar graph illustrating the Western blot results for Caspase-3 and cleaved Caspase-3 expression in Panc02 cells treated with rAj-HRP30. (**h**) Bar graph illustrating the Western blot results for PARP1 and cleaved PARP1 expression in Panc02 cells treated with rAj-HRP30. (**i**) Statistical analysis of the relative optical densities of Caspase-9 and cleaved Caspase-9 (target proteins) normalized to GAPDH levels. (**j**) Statistical analysis of the relative optical densities of Caspase-7 and cleaved Caspase-7 (target proteins) normalized to GAPDH levels. (**k**) Statistical analysis of the relative optical densities of Caspase-3 and cleaved Caspase-3 (target proteins) normalized to GAPDH levels. (**l**) Statistical analysis of the relative optical densities of PARP1 and cleaved PARP1 (target proteins) normalized to GAPDH levels. (*n* = 3); the control group was treated with a blank medium, and the experimental groups were treated with rAj-HRP30. Significant differences between the blank group and the experimental groups are expressed by * *p* < 0.05, ** *p* < 0.01, *** *p* < 0.001, and **** *p* < 0.0001.

Bcl-2 proteins primarily function by modulating apoptotic signaling pathways, maintaining the redox state of cells through a series of mechanisms that inhibit oxidative reactions, thereby preventing apoptosis [25]. Within this family, Bax can interact with Bcl-2 to form homodimers that promote apoptosis or heterodimers with other proteins that inhibit apoptotic processes [26]. As such, the relative expression levels of pro-apoptotic and anti-apoptotic proteins serve as key indicators of whether apoptosis will occur, with the Bax/Bcl-2 ratio functioning as a “molecular switch” for cell death [27]. In the present study, the impact of rAj-HRP30 on the expression levels of Bax and Bcl-2 in Panc02 cells was assessed. The experimental results (Figure 9a–d) demonstrate that, as the concentration of rAj-HRP30 increased, the expression of Bax and Bcl-2 exhibited inverse trends; the relative expression of Bcl-2 progressively decreased, while that of Bax increased. Consequently, the Bax/Bcl-2 ratio also declined, a trend that was both concentration-dependent and statistically significant. These findings suggest that rAj-HRP30 effectively downregulates the Bax/Bcl-2 ratio, thereby facilitating the activation of apoptotic signaling pathways mediated by the Bcl-2 family.

Caspases are cytoplasmic enzymes with cysteine-containing active sites that regulate programmed cell death, including apoptosis and necroptosis, by selectively cleaving and inactivating target proteins [28]. This study assessed the effect of rAj-HRP30 on the expression of Caspase-9, -3, -7, and their cleaved forms. The experimental results (Figure 9e–g,i–k) indicate that, as the concentration of rAj-HRP30 increased, the proenzymes of Caspase-9, Caspase-7, and Caspase-3 were progressively degraded, with a corresponding increase in the cleaved products, including cleaved Caspase-9, cleaved Caspase-7, and cleaved Caspase-3. These findings suggest that rAj-HRP30 induces the homologous activation of Caspase-9, Caspase-7, and Caspase-3 in Panc02 cells, leading to the production of their cleaved forms and the subsequent initiation of a caspase cascade, ultimately resulting in apoptosis.

PARP-1 plays a critical role in the repair of damaged DNA and is also significantly involved in the process of apoptosis. This study assessed the effect of rAj-HRP30 on the expression of PARP-1 and its cleaved form. The experimental results (Figure 9h,l) demonstrate that, as the concentration of rAj-HRP30 increased, there was an enhanced degradation of the proenzyme PARP-1, leading to a corresponding increase in the cleaved product, cleaved PARP-1. These findings suggest that rAj-HRP30 induces the homologous activation of PARP-1 in Panc02 cells, resulting in the generation of cleaved PARP-1 and the subsequent induction of apoptosis.

### 2.7. rAj-HRP30 Acts on FGFR1 Targets to Inhibit Downstream Signaling Pathways

**Figure 10:**
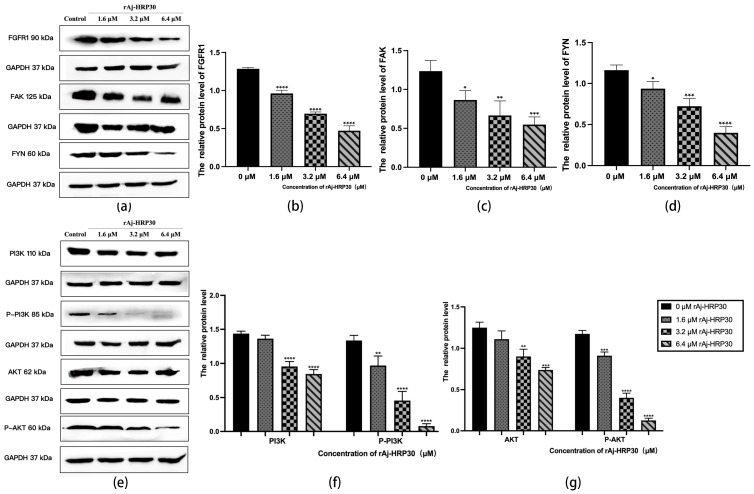
(**a**) Bar graph illustrating the Western blot results for FGRF1, FYN, and FAK expression in Panc02 cells treated with rAj-HRP30. (**b**) Statistical analysis of the relative optical densities of FGRF1 (target proteins) normalized to GAPDH levels (*n* = 3); the control group was treated with a blank medium, and the experimental groups were treated with rAj-HRP30. Significant differences between the blank group and the experimental groups are expressed by **** *p* < 0.0001. (**c**) Statistical analysis of the relative optical densities of FAK (target proteins) normalized to GAPDH levels (*n* = 3); the control group was treated with a blank medium, and the experimental groups were treated with rAj-HRP30. Significant differences between the blank group and the experimental groups are expressed by * *p* < 0.05, ** *p* < 0.01, and *** *p* < 0.001. (**d**) Statistical analysis of the relative optical densities of FYN (target proteins) normalized to GAPDH levels (*n* = 3); the control group was treated with a blank medium, and the experimental groups were treated with rAj-HRP30. Significant differences between the blank group and the experimental groups are expressed by * *p* < 0.05, *** *p* < 0.001, and **** *p* < 0.0001. (**e**) Bar graph illustrating the Western blot results for PI3K, p-PI3K, AKT, and p-AKT expression in Panc02 cells treated with rAj-HRP30. (**f**) Statistical analysis of the relative optical densities of PI3K and p-PI3K (target proteins) normalized to GAPDH levels (*n* = 3); the control group was treated with a blank medium, and the experimental groups were treated with rAj-HRP30. Significant differences between the blank group and the experimental groups are expressed by ** *p* < 0.01 and **** *p* < 0.0001. (**g**) Statistical analysis of the relative optical densities of AKT and p-AKT (target proteins) normalized to GAPDH levels (*n* = 3); the control group was treated with a blank medium, and the experimental groups were treated with rAj-HRP30. Significant differences between the blank group and the experimental groups are expressed by ** *p* < 0.01, *** *p* < 0.001, and **** *p* < 0.0001.

Fibroblast growth factor receptors (FGFRs) are tyrosine kinase receptors in the receptor tyrosine kinase (RTK) family. The human genome encodes four FGFR subtypes, each with extracellular, transmembrane, and intracellular domains containing phosphorylation sites. Ligand binding triggers dimerization and autophosphorylation, activating the PI3K/AKT signaling pathway. FGFR signaling regulates cell growth, differentiation, angiogenesis, migration, organ development, and wound healing [29]. In this study, the effect of rAj-HRP30 on FGFR1 expression in Panc02 cells was assessed. The results (Figure 10a,b) demonstrate a concentration-dependent decrease in the relative expression of FGFR1, with a progressive decline observed as the concentration of rAj-HRP30 increased. Based on these findings, we hypothesize that rAj-HRP30 may downregulate FGFR1 expression, thereby inhibiting the activation of the FGFR1 signaling pathway.

Focal adhesion kinase (FAK) is a cytoplasmic non-receptor tyrosine kinase that responds to biological and mechanical signals. It regulates cell growth and survival by activating the PI3K/AKT and Ras/MAPK pathways. FAK also plays a vital role in embryonic development, tumor formation, and cell migration [30]. This study assessed the effect of rAj-HRP30 on FAK expression in Panc02 cells. The experimental results (Figure 10a,c) reveal a concentration-dependent decrease in FAK expression with increasing rAj-HRP30 concentrations. Based on these findings, it is hypothesized that rAj-HRP30 may inhibit FAK expression, thereby suppressing the activation of the FAK signaling pathway.

Fyn tyrosine kinase (FYN), a key Src family kinase (SFK), drives tumor development and progression by regulating cell adhesion, invasion, proliferation, survival, apoptosis, and angiogenesis. It also modulates cancer-related signaling pathways, including ERK, COX-2, STAT5, MET, and AKT [31]. This study assessed the effect of rAj-HRP30 on FAK expression in Panc02 cells. The results (Figure 10a,d) demonstrate a gradual decrease in the relative expression of FYN as the concentration of rAj-HRP30 increased, showing a concentration-dependent effect. This suggests that rAj-HRP30 may suppress the activation of the FYN signaling pathway by downregulating FYN expression.

The PI3K/AKT pathway regulates vital cellular functions such as survival, migration, metabolism, angiogenesis, and inflammation. It is essential for physiological processes and contributes to tumor development and progression. PI3K phosphorylates PIP2 to produce PIP3, which recruits downstream molecules, including AKT [32]. In this study, we assessed the effect of rAj-HRP30 on the expression of PI3K, p-PI3K, AKT, and p-AKT in Panc02 cells. The experimental results (Figure 10e,f) indicate that, with increasing concentrations of rAj-HRP30, the relative expression levels of PI3K and p-PI3K gradually decreased, suggesting that rAj-HRP30 may downregulate the expression of PI3K while promoting its phosphorylation, leading to a significant reduction in p-PI3K expression. This implies that rAj-HRP30 may inhibit the activation of the PI3K/AKT pathway through the downregulation of PI3K. Furthermore, the data from Figure 10g reveal that the relative expression levels of AKT and p-AKT progressively decreased with increasing rAj-HRP30 concentrations in a dose-dependent manner, indicating that rAj-HRP30 can suppress AKT expression and affect its phosphorylation status, thereby inhibiting the activation of the PI3K/AKT signaling pathway.

## 3. Discussion

In this study, we began with the wild-type rAj-HRP, a histidine-rich peptide derived from the sea cucumber *Apostichopus japonicus*, and designed and synthetically constructed its truncated mutant variant, rAj-HRP30. After successful cloning and expression, the recombinant rAj-HRP30 was purified using nickel ion affinity chromatography. Bioinformatics analysis revealed that rAj-HRP30 shares over 68% homology with HRG-like genes from various species, including *Spodoptera litura*, *Plasmodium lophurae*, *Bicyclus anynana*, and *Pararge aegeria*. While the functional characterization of HRG-like proteins in these species remains unreported, substantial research has been conducted on the human histidine-rich glycoprotein (HRG). Human HRG is a multi-domain plasma glycoprotein that binds a variety of ligands, including zinc ions, plasminogen, and thrombin-sensitive peptides [33]. Due to its ability to interact with multiple ligands, HRG exhibits a broad range of biological activities, such as regulating angiogenesis, promoting the clearance of immune complexes, modulating coagulation and fibrinolysis, inhibiting tumor proliferation, inducing apoptosis, acting as a pH sensor and zinc ion detector, and demonstrating antimicrobial properties [21,22]. Among these diverse functions, the antitumor activity of human HRG has garnered particular attention. Based on this context, we sought to explore the key scientific question of whether the sea cucumber-derived peptide rAj-HRP30 also possesses antitumor properties, which we aimed to validate experimentally.

In selecting a tumor cell model for our study, we chose pancreatic cancer cells, one of the top ten most malignant cancers. The primary activities of malignant tumor cells, including proliferation, adhesion, migration, and invasion, contribute to both the growth and metastasis of primary tumors. Therefore, our in vitro experiments aimed to replicate these biological processes to assess the effect of rAj-HRP30 on tumor cells. The experimental results demonstrated that rAj-HRP30 significantly inhibited the proliferation, adhesion, migration, and invasion of Panc01 and Panc02 cells. Additionally, it induced apoptosis and cytoskeletal depolymerization in these cells. These findings clearly indicate that the recombinant sea cucumber peptide rAj-HRP30 exhibits antitumor biological activity akin to that of human HRG, with promising results in the Panc01 and Panc02 cell models. However, the in vivo antitumor activity of rAj-HRP30 remains to be validated, and further investigations are required to assess whether it retains similar inhibitory effects in tumor cells in vivo.

In the field of oncology, the dysregulation of the fibroblast growth factor (FGF)/FGF receptor (FGFR) family is widely recognized as a critical contributor to carcinogenesis, tumor progression, and resistance to cancer therapies across various tumor types. The epidermal growth factor receptor (FGFR) family consists of four highly conserved transmembrane receptor tyrosine kinases (FGFR1-4). The aberrant activation of these receptors can trigger multiple cancer-associated signaling pathways, such as MAPK, PLCγ, PI3K/AKT, and JAK/STAT, among others. The activation of these pathways ultimately accelerates the malignant transformation of cancer. Studies have shown that cancer cells treated with epidermal growth factor receptor inhibitors often exhibit increased sensitivity to anticancer drugs, further underscoring the pivotal role of FGF/FGFR signaling in cancer stem cell regulation and highlighting its potential as a therapeutic target. According to the literature, the protein domains of FGF include the FGF receptor (FGFR) binding domain and the heparin sulfate (HS) binding domain, both of which play crucial roles in the dimerization of FGFR [34]. FGF initially binds to HS, which then facilitates its interaction with FGFR, thereby activating the FGF signaling pathway [35,36,37]. Nearly all FGFs require HS as a cofactor to activate FGFR. Moreover, human histidine-rich glycoprotein (HRG) can competitively bind to the HS ligand of FGF, thereby inhibiting the activation of the FGF signaling pathway [38,39]. The conclusion drawn in this chapter—that the sea cucumber-derived histidine-rich peptide rAj-HRP30 downregulates FGFR expression—is highly consistent with the existing literature regarding the inhibitory mechanism of human HRG on the FGF pathway. Additionally, functional studies showing that rAj-HRP30 inhibits bFGF-induced cell migration and invasion further substantiate its inhibitory effect on FGF signaling. Based on these findings, it is hypothesized that the inhibitory action of rAj-HRP30 may stem from its competitive binding to the HS ligand of FGF, thereby preventing the activation of the FGF signaling pathway.

Supported by this theoretical framework, the present study demonstrated that rAj-HRP30 significantly downregulates the expression of FGFR, thereby inhibiting its downstream signaling pathways, including FYN and FAK, ultimately suppressing the activation of the PI3K/AKT pathway. Within the PI3K/AKT signaling cascade, rAj-HRP30 downregulates the expression of PI3K, p-PI3K, AKT, and p-AKT in Panc02 cells, further activating the mitochondrial apoptotic signaling pathway. This activation is evidenced by the upregulation of Bax expression and the downregulation of Bcl-2 expression, promoting the activation of downstream molecules and inducing apoptosis. Notably, assays targeting key molecules revealed that rAj-HRP30 significantly enhances the degradation of caspase precursors, including Caspase-9, Caspase-3, Caspase-7, and PARP1, resulting in the generation of more cleaved products, as indicated by the upregulation of cleaved Caspase-9, cleaved Caspase-3, cleaved Caspase-7, and cleaved PARP1, with a distinct concentration-dependent characteristic. Additionally, rAj-HRP30 markedly downregulates the expression of MMP2 and MMP9 in Panc02 cells, thereby inhibiting their migratory capacity.

## 4. Materials and Methods

### 4.1. rAj-HRP30 Gene Design, Synthesis, and Cloning

Based on the sequence analysis of rAj-HRP, we selected the amino acid sequence from a position that could encode the desired peptide fragment as our target shortened peptide, which we designated rAj-HRP30. After optimizing the codons for these 30 amino acids, we chose *Nde* I and *Xho* I as the cloning sites. Additionally, to eliminate the vector purification tag, a stop codon was added to the 3′ end of the rAj-HRP30 gene. The gene and primer sequences were synthesized by GenScript (Nanjing, China). The primer sequences are as follows:

5′-primer (P1):

5′-XXCATATGAGGCACAGACACGGATGG-3′, containing the *Nde* I restriction enzyme site.

3′-primer (P2):

5′-XXCTCGAGTTAGTGGTGCTCGCCGTGGTG-3′, containing the *Xho* I restriction enzyme site.

Using the synthetic rAj-HRP30 gene as a template, PCR amplification was performed with the primers described above. The resulting product was subjected to double digestion with *Nde* I and *Xho* I, followed by ligation into the pET23b plasmid vector using T4 DNA ligase. The recombinant plasmid, pET23b-HRP30, was subsequently verified through double digestion (carried out by GenScript, Nanjing, China). Sequence alignment of rAj-HRP30 was conducted via the NCBI website.

### 4.2. Induced Expression and Purification of rAj-HRP30

This study aimed to utilize the CaCl_2_-mediated transformation method to introduce the previously constructed recombinant plasmid pET23b-HRP30 into the *Escherichia coli* BL21 expression strain, followed by IPTG-induced protein expression. After ultrasonic disruption of the expressed cells, the recombinant protein was purified using a nickel ion affinity chromatography column. Subsequently, tricine SDS-PAGE electrophoresis was employed to analyze the molecular weight of the purified protein. Finally, the concentration of the recombinant sea cucumber peptide rAj-HRP30 was determined using a Coomassie Brilliant Blue G-250 assay.

### 4.3. Cell Culture

The human pancreatic ductal adenocarcinoma Panc01 cells and mouse pancreatic ductal adenocarcinoma Panc02 cells were both obtained from the Cell Bank of the Chinese Academy of Sciences. In this study, the Panc01 cells were cultured in DMEM complete medium (DMEM/FBS = 900:100), while the Panc02 cells were cultured in RPMI 1640 complete medium (RPMI 1640/FBS/Pen Strep = 888:100:12). The cells were maintained under standard culture conditions of 37 °C with 5% CO_2_.

### 4.4. Cell Proliferation Assay

In a 96-well plate, 100 μL of cell suspension was added to each well, with a typical cell density ranging from 3 × 10^3^ to 5 × 10^3^ cells per well. Based on preliminary experiments, rAj-HRP30 was added to the Panc01 experimental wells at concentrations ranging from 0.78 to 7.02 μM and to the Panc02 experimental wells at concentrations ranging from 1.18 to 10.60 μM. Each experimental group included three replicate wells and the volumes in each well were adjusted to ensure consistent final volumes using DMEM or RPMI 1640 culture medium. The plate was then incubated in a cell culture incubator for 22 to 26 h. After the incubation period, and in the absence of light, 10 μL of CCK-8 solution (Sangon Biotech, Shanghai, China) was added to each well. The wells were wrapped in aluminum foil and incubated for an additional 4 h. Finally, absorbance at 450 nm was measured using a microplate reader (TECAN, Männedorf, Switzerland), and the average values of the three replicates were calculated.

### 4.5. Cell Morphology Experiment

The Panc01 and Panc02 cell suspensions (at a concentration of 70%) were each seeded at 500 μL per well in two 24-well plates pre-coated with cell coverslips. After gentle shaking to ensure even distribution, the plates were incubated for 24 h. Following this incubation, the original culture medium was removed, and fresh medium was added, along with varying concentrations of rAj-HRP30 (for the Panc01 cell plate, final concentrations of 0, 2.75, 5.5, and 8.25 μM were applied; for the Panc02 cell plate, final concentrations of 0, 1.6, 3.2, and 6.4 μM were used). The cells were then cultured for an additional 24 h. Subsequently, staining was performed according to the manufacturer’s instructions for Giemsa stain (Solarbio, Beijing, China). The coverslips were observed under an inverted microscope, and experimental observations were captured through imaging.

### 4.6. Experiments on Cell Adhesion Capacity

After preparing two pre-cooled 96-well plates, 40 μL of four adhesion factors (COL, VN, LN, and FN) at a final concentration of 0.1 μg/μL was added to each well. The plates were then wrapped in aluminum foil and incubated overnight at 4 °C. Following the removal of the adhesion molecules, Panc01 and Panc02 cell suspensions were seeded into the two 96-well plates at a volume of 100 μL per well and treated with varying concentrations of rAj-HRP30 for 4 h. Subsequently, in a light-protected environment, 10 μL of CCK-8 solution was added to each well, and the plates were wrapped in aluminum foil and incubated for an additional 4 h. Finally, the absorbance at 450 nm was measured using a microplate reader (TECAN, Männedorf, Switzerland). The cell adhesion rate was calculated using the formula:

$$adhesion\;rate=\frac{OD\;value\;of\;experimental\;group}{OD\;value\;of\;control\;group}\times 100\%$$

### 4.7. Experiments on the Migratory Capacity of Cells

Before initiating the experiment, the basal membrane of the Transwell chambers was hydrated, and the Panc01 and Panc02 cells were cultured in a serum-free medium for 12 h to induce serum starvation. Subsequently, serum-free cell suspensions were prepared. In the lower chambers of the Transwell plates, 600 μL of pre-warmed DMEM medium containing bFGF, RMPI 1640 medium, and DMEM and RMPI 1640 media lacking bFGF was added. In the upper chambers, 100 μL of the mixed Panc01 and Panc02 cell suspensions was introduced, and the cells were treated with varying concentrations of rAj-HRP30 for 34 to 36 h. Upon completion of the treatment, the medium was removed, and non-migratory cells were gently wiped away using sterile cotton swabs. Subsequently, 4% paraformaldehyde fixative was added to the lower chambers, and the cells were fixed at room temperature for 30 min. Finally, the cells were stained according to the instructions for Crystal Violet staining solution (Sangon Biotech, Shanghai, China). The migratory cells were observed under an inverted microscope, and images were captured to document experimental phenomena. Cell counts were performed using ImageJ2 2.9.0/1.53t software (Rawak Software Inc., Stuttgart, Germany), and the inhibitory effect of rAj-HRP30 on Panc01 and Panc02 cell migration was calculated. The formula is as follows:

$$\begin{array}{ll} Cell\;migration & inhibition\;rate\\ & =\frac{Number\;of\;cells\;in\;the\;bFGF\;control\;group-Number\;of\;cells\;in\;experimental\;group}{Number\;of\;cells\;in\;the\;bFGF\;control\;group}\\ & \times 100\% \end{array}$$

### 4.8. Experiments on the Invasive Capacity of Cells

Transwell plates, 200 μL sterile pipette tips, and Matrigel matrix (at a concentration of 2 mg/mL, diluted appropriately with DMEM and RPMI 1640 media) were pre-chilled at 4 °C. Concurrently, Panc01 and Panc02 cells were serum-starved for 12 h in a serum-free medium, and cell suspensions were subsequently prepared in a serum-free medium. The pre-chilled Transwell plates were slowly coated with 40 μL/well of diluted Matrigel matrix in the upper chambers, which were then incubated in a cell culture incubator for 0.5 to 1 h until the Matrigel formed a membrane-like structure, after which the plates were removed. Next, 600 μL/well of pre-warmed DMEM and RPMI 1640 media containing bFGF, along with DMEM and RPMI 1640 media without bFGF, was added to the lower chambers of the Transwell plates. To the upper chambers, 100 μL of the well-mixed Panc01 and Panc02 cell suspensions was added, along with different concentration gradients of rAj-HRP30. The cells were then incubated for 34 to 36 h. After the incubation period, the media were removed, and non-migrated cells were gently wiped off using a sterile cotton swab. Subsequently, 4% paraformaldehyde fixative was added to the lower chamber, and the cells were fixed at room temperature for 30 min. Finally, staining was performed according to the instructions provided for Crystal Violet staining solution (Sangon Biotech, Shanghai, China). The cell invasion was visualized using an inverted microscope, and images were captured to document the experimental observations. Cell counting was conducted using ImageJ2 2.9.0/1.53t software (Rawak Software Inc., Stuttgart, Germany), and the inhibition rate of rAj-HRP30 on Panc01 and Panc02 cell invasion was calculated.

### 4.9. Cytofluorescence Assay

A 500 µL aliquot of the Panc01 and Panc02 cell suspensions (with a suspension density of 70%) was seeded into 24-well plates pre-coated with cell culture slides. After the suspensions were gently mixed using a cross-pattern motion, the plates were incubated in a cell culture incubator for 24 h. Following the incubation, the original medium was replaced with fresh medium, and the cells were treated with rAj-HRP30 at various concentrations for 24 h. After treatment, the cells were fixed with 4% paraformaldehyde for 30 min. Subsequently, staining was performed according to the instructions provided for Hoechst staining solution, TUNEL reagent, and FITC Phalloidin working solution (Beyotime, Shanghai, China). Upon completion of the staining, the cell culture slides were removed under light-protected conditions, inverted, and placed onto sterile glass slides with a drop of antifade mounting medium. Fluorescence microscopy was employed to observe the experimental phenomena and capture images for documentation.

### 4.10. Western Blot Assay

Panc02 cells treated with various concentrations of rAj-HRP30 were collected into centrifuge tubes and centrifuged at 12,000 rpm for 5 min at 4 °C. The cells were then resuspended in cell lysis buffer and mixed thoroughly, followed by the addition of 5× loading buffer to the centrifuge tube. A volume of 20 µL per well of the sample was loaded into pre-prepared SDS-PAGE gels, and electrophoresis was carried out until the bromophenol blue dye reached the bottom of the gel, at which point the running was stopped. Following protein separation based on molecular weight, the proteins were transferred onto a PVDF membrane. The membrane was subsequently blocked with a 5% non-fat milk-blocking solution on a shaking platform for 3 h. After blocking, the PVDF membrane (Millipore, Darmstadt, Germany) was incubated overnight at 4 °C in an antibody-containing incubation bag. The membrane was then washed four times with TBST buffer at room temperature, each wash lasting 10 min. Finally, the PVDF membrane was treated with an ECL chemiluminescent substrate (Beyotime, Shanghai, China), and the signals were detected using a chemiluminescent gel imaging system (Bio-Rad, Hercules, CA, USA).

The antibodies used in this study were as follows: anti-GAPDH (Cell Signaling Technology, Danvers, MA, USA, 1:4000), anti-FGFR1 (Cell Signaling Technology, Danvers, MA, USA, 1:1000), anti-FYN (Cell Signaling Technology, Danvers, MA, USA, 1:20,000), anti-FAK (Cell Signaling Technology, Danvers, MA, USA, 1:1000), anti-AKT (Cell Signaling Technology, Danvers, MA, USA, 1:1000), anti-phospho-AKT (Cell Signaling Technology, Danvers, MA, USA, 1:1000), anti-PI3K (Cell Signaling Technology, Danvers, MA, USA, 1:1000), anti-phospho-PI3K (Cell Signaling Technology, Danvers, MA, USA, 1:1000), anti-BAX (Proteintech, Chicago, IL, USA, 1:5000), anti-Bcl2 (Proteintech, Chicago, IL, USA, 1:2000), anti-MMP2 (Proteintech, Chicago, IL, USA, 1:800), anti-MMP9 (Proteintech, Chicago, IL, USA, 1:600), anti-PARP (Proteintech, Chicago, IL, USA, 1:2000), anti-Caspase-3/p17/p19 (Cell Signaling Technology, Danvers, MA, USA, 1:1000), anti-Caspase-7/p20 (Proteintech, Chicago, IL, USA, 1:600), and anti-Caspase-9/p35/p10 (Proteintech, Chicago, IL, USA, 1:300). Horseradish peroxidase-conjugated goat anti-rabbit (ZSGB-BIO, Beijing, China, 1:5000) and horseradish peroxidase-conjugated goat anti-mouse (ZSGB-BIO, Beijing, China, 1:5000) were used as secondary antibodies.

## 5. Conclusions

In this study, the recombinant sea cucumber peptide rAj-HRP30 was successfully expressed in *Escherichia coli* BL21 and purified via nickel ion affinity chromatography, resulting in a concentration of 0.526 μg/µL, a purity of 95%, and a protein yield of 0.723 mg/L. The experimental results revealed that rAj-HRP30 significantly inhibited the in vitro activity of both human- and mouse-derived pancreatic ductal adenocarcinoma (PDAC) cell lines, Panc01 and Panc02, in a dose-dependent manner.

Specifically, rAj-HRP30 suppressed cell proliferation, with IC50 values of 5.54 μM and 3.22 μM for Panc01 and Panc02 cells, respectively. Furthermore, rAj-HRP30 reduced the adhesion of these cells to extracellular matrix components, including VN, FN, LN, and COL, with notable decreases observed at higher doses. Additionally, the peptide inhibited the migration and invasion of both cell lines under bFGF stimulation, with inhibition rates reaching up to 90% at high concentrations.

Moreover, rAj-HRP30 induced apoptosis in Panc01 and Panc02 cells, as evidenced by morphological changes and cytoskeletal depolymerization. This was accompanied by the activation of key apoptotic markers, including Caspase-9, -3, -7, and cleaved PARP1, along with the downregulation of anti-apoptotic proteins such as Bcl-2. The observed inhibitory effects of rAj-HRP30 on PDAC cell activity were closely linked to the FGFR signaling and apoptosis pathways, suggesting that FGFR is a potential target for this peptide.

In conclusion, rAj-HRP30 demonstrates promising therapeutic potential as a novel anticancer agent for pancreatic cancer, with its multi-faceted mechanism of action involving the inhibition of proliferation, adhesion, migration, and invasion and the induction of apoptosis in PDAC cells. Future studies should further investigate the underlying molecular interactions and explore the in vivo efficacy of rAj-HRP30 to validate its potential for clinical application.

## Data Availability

The data presented in this study are available on request from the corresponding author. The data are not publicly available, and due to our lab’s policies or confidentiality agreements, we cannot provide the raw data.
